# 2-(3-Pyridinio)benzimidazolium penta­chloridobismuthate(III) monohydrate

**DOI:** 10.1107/S1600536809024568

**Published:** 2009-07-01

**Authors:** Li-Jing Cui, Hai-Jun Xu, Yi-Jie Pang

**Affiliations:** a Ordered Matter Science Research Center, College of Chemistry and Chemical Engineering, Southeast University, Nanjing 211189, People’s Republic of China

## Abstract

In the title compound, (C_12_H_11_N_3_)[BiCl_5_]·H_2_O, the Bi^III^ atom is coordinated by five chloride anions in a distorted square-pyramidal geometry. The planar imidazole ring system [maximum deviation = 0.012 (3) Å] is oriented at a dihedral angle of 6.08 (5)° with respect to the protonated pyridine ring. An O—H⋯Cl inter­action links the water mol­ecule to the dianion. In the crystal structure, inter­molecular O—H⋯Cl, N—H⋯O and N—H⋯Cl inter­actions link the mol­ecules into a three-dimensional network.

## Related literature

For the properties of bis­muthate(III) compounds, see: Turel *et al.* (1998 ▶); Goforth *et al.* (2004 ▶).
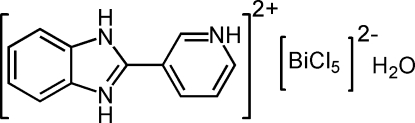


## Experimental

### 

#### Crystal data

(C_12_H_11_N_3_)[BiCl_5_]·H_2_O
*M* *_r_* = 601.48
Monoclinic, 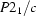

*a* = 9.3297 (19) Å
*b* = 13.391 (3) Å
*c* = 14.476 (3) Å
β = 101.78 (3)°
*V* = 1770.6 (6) Å^3^
*Z* = 4
Mo *K*α radiation
μ = 10.72 mm^−1^
*T* = 294 K
0.25 × 0.20 × 0.16 mm

#### Data collection

Rigaku SCXmini diffractometer
Absorption correction: multi-scan (*CrystalClear*; Rigaku, 2005 ▶) *T*_min_ = 0.090, *T*_max_ = 0.180
17812 measured reflections
4049 independent reflections
2822 reflections with *I* > 2σ(*I*)
*R*_int_ = 0.115

#### Refinement

*R*[*F* ^2^ > 2σ(*F* ^2^)] = 0.052
*wR*(*F* ^2^) = 0.116
*S* = 1.15
4049 reflections
199 parameters
H-atom parameters constrained
Δρ_max_ = 1.28 e Å^−3^
Δρ_min_ = −2.37 e Å^−3^

### 

Data collection: *CrystalClear* (Rigaku, 2005 ▶); cell refinement: *CrystalClear*; data reduction: *CrystalClear*; program(s) used to solve structure: *SHELXS97* (Sheldrick, 2008 ▶); program(s) used to refine structure: *SHELXL97* (Sheldrick, 2008 ▶); molecular graphics: *SHELXTL* (Sheldrick, 2008 ▶); software used to prepare material for publication: *SHELXL97*.

## Supplementary Material

Crystal structure: contains datablocks I, global. DOI: 10.1107/S1600536809024568/hk2717sup1.cif

Structure factors: contains datablocks I. DOI: 10.1107/S1600536809024568/hk2717Isup2.hkl

Additional supplementary materials:  crystallographic information; 3D view; checkCIF report

## Figures and Tables

**Table 1:** Hydrogen-bond geometry (Å, °)

*D*—H⋯*A*	*D*—H	H⋯*A*	*D*⋯*A*	*D*—H⋯*A*
N1—H1*A*⋯Cl5^i^	0.86	2.65	3.267 (8)	130
N1—H1*A*⋯Cl1^ii^	0.86	2.66	3.302 (8)	133
N2—H2*A*⋯O1*W*^iii^	0.86	1.86	2.680 (9)	159
N3—H3*B*⋯Cl4^iv^	0.86	2.33	3.104 (9)	150
O1*W*—H1*WA*⋯Cl3^ii^	0.85	2.62	3.292 (7)	137
O1*W*—H1*WB*⋯Cl5	0.85	2.77	3.190 (7)	112

Symmetry codes: (i) 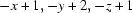; (ii) 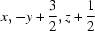; (iii) 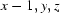; (iv) 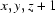.

**Fig. 1.**
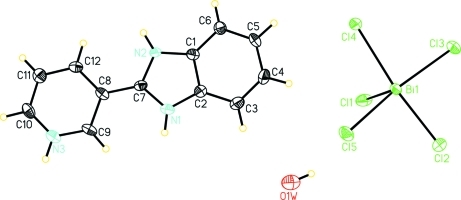
The molecular structure of the title molecule, with the atom-numbering scheme. Displacement ellipsoids are drawn at the 30% probability level

**Fig. 2.**
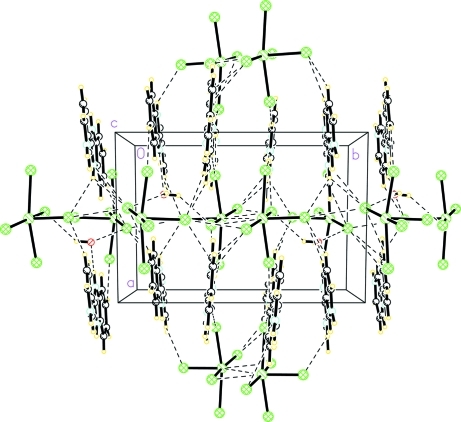
A partial packing diagram.

## supplementary crystallographic information

### Comment

Bismuthate(III) compounds have received great attention owing to their anti-ulcer activity (Turel *et al.*, 1998) and their unique optical and electronic properties, including nonlinear optical activity, luminescence and semiconductivity (Goforth *et al.*, 2004). We report herein the crystal structure of the title compound.

The asymmetric unit of the title compound, (Fig. 1), contains a 2-(3'-pyridinio)benzimidazolium dication, a pentachlorobismuthate dianion and a water molecule. In the dianion, the bismuth (III) atom is coordinated by five chloride anions in a distorted square-pyramidal geometry. The Bi-Cl distances are in the range of 2.519 (3)-2.787 (3) Å. In the dication, the planar imidazole ring system [with maximum deviation of 0.012 (3) Å for atom C3] is oriented with respect to the pyridine ring at a dihedral angle of 6.08 (5)°. Intramolecular O-H···Cl interaction links the water molecule to the dianion (Table 1).

In the crystal structure, intramolecular O-H···Cl and intermolecular N-H···O and N-H···Cl interactions (Table 1) link the molecules into a three-dimensional network (Fig. 2), in which they may be effective in the stabilization of the structure.

### Experimental

For the preparation of the title compound, concentrated hydrochloric acid (12 M) was added dropwise to a mixture of 2-(3-pyridinio)benzimidazole (0.1 mmol) and water (7 ml), until complete dissolution of the solid phase. Concentrated hydrochloric acid was similarly added dropwise to dissolve the solid phase persisting in a mixture of bismuth chloride (0.3 mmol) and water (5 ml). The two solutions were then mixed and stirred for 20 min. The resulting precipitate was filtered off and dissolved in hydrochloric acid. Colorless crystals suitable for X-ray analysis were obtained after several weeks by slow evaporation of the solvent at room temperature.

### Refinement

H atoms were positioned geometrically with O-H = 0.85 Å (for H_2_O), N-H = 0.86 Å (for NH) and C-H = 0.93 Å for aromatic H atoms and constrained to ride on their parent atoms, with U_iso_(H) = 1.2U_eq_(C,N,O).

### Crystal data

**Table:**

(C_12_H_11_N_3_)[BiCl_5_]·H_2_O	*F*(000) = 1128
*M**_r_* = 601.48	*D*_x_ = 2.256 Mg m^−^^3^
Monoclinic, *P*2_1_/*c*	Mo *K*α radiation, λ = 0.71073 Å
Hall symbol: -P 2ybc	Cell parameters from 1647 reflections
*a* = 9.3297 (19) Å	θ = 3.0–27.6°
*b* = 13.391 (3) Å	µ = 10.72 mm^−^^1^
*c* = 14.476 (3) Å	*T* = 294 K
β = 101.78 (3)°	Prism, colorless
*V* = 1770.6 (6) Å^3^	0.25 × 0.20 × 0.16 mm
*Z* = 4	

### Data collection

**Table:**

Rigaku SCXmini diffractometer	4049 independent reflections
Radiation source: fine-focus sealed tube	2822 reflections with *I* > 2σ(*I*)
graphite	*R*_int_ = 0.115
Detector resolution: 13.6612 pixels mm^-1^	θ_max_ = 27.5°, θ_min_ = 3.0°
ω scans	*h* = −12→12
Absorption correction: multi-scan (*CrystalClear*; Rigaku, 2005)	*k* = −17→17
*T*_min_ = 0.090, *T*_max_ = 0.180	*l* = −18→18
17812 measured reflections	

### Refinement

**Table:**

Refinement on *F*^2^	Primary atom site location: structure-invariant direct methods
Least-squares matrix: full	Secondary atom site location: difference Fourier map
*R*[*F*^2^ > 2σ(*F*^2^)] = 0.052	Hydrogen site location: inferred from neighbouring sites
*wR*(*F*^2^) = 0.116	H-atom parameters constrained
*S* = 1.15	*w* = 1/[σ^2^(*F*_o_^2^) + (0.0236*P*)^2^] where *P* = (*F*_o_^2^ + 2*F*_c_^2^)/3
4049 reflections	(Δ/σ)_max_ < 0.001
199 parameters	Δρ_max_ = 1.28 e Å^−^^3^
0 restraints	Δρ_min_ = −2.37 e Å^−^^3^

### Special details

**Table:**

Geometry. All e.s.d.'s (except the e.s.d. in the dihedral angle between two l.s. planes) are estimated using the full covariance matrix. The cell e.s.d.'s are taken into account individually in the estimation of e.s.d.'s in distances, angles and torsion angles; correlations between e.s.d.'s in cell parameters are only used when they are defined by crystal symmetry. An approximate (isotropic) treatment of cell e.s.d.'s is used for estimating e.s.d.'s involving l.s. planes.
Refinement. Refinement of *F*^2^ against ALL reflections. The weighted *R*-factor *wR* and goodness of fit *S* are based on *F*^2^, conventional *R*-factors *R* are based on *F*, with *F* set to zero for negative *F*^2^. The threshold expression of *F*^2^ > σ(*F*^2^) is used only for calculating *R*-factors(gt) *etc*. and is not relevant to the choice of reflections for refinement. *R*-factors based on *F*^2^ are statistically about twice as large as those based on *F*, and *R*- factors based on ALL data will be even larger.

### Fractional atomic coordinates and isotropic or equivalent isotropic displacement parameters (Å^2^)

**Table:**

	*x*	*y*	*z*	*U*_iso_*/*U*_eq_	
Bi1	0.50077 (4)	0.91031 (3)	0.12159 (2)	0.03242 (14)	
Cl1	0.4928 (3)	0.7336 (2)	0.1799 (2)	0.0538 (8)	
Cl2	0.7784 (3)	0.8830 (2)	0.1188 (2)	0.0516 (8)	
Cl3	0.4576 (3)	0.8682 (2)	−0.07062 (17)	0.0432 (6)	
Cl4	0.1991 (3)	0.8994 (2)	0.09655 (18)	0.0461 (7)	
Cl5	0.5648 (3)	0.9799 (2)	0.29758 (18)	0.0474 (7)	
O1W	0.6526 (7)	0.8372 (5)	0.4766 (5)	0.060 (2)	
H1WA	0.6470	0.7747	0.4854	0.072*	
H1WB	0.6594	0.8234	0.4204	0.072*	
N1	0.1677 (8)	0.8703 (6)	0.6102 (6)	0.041 (2)	
H1A	0.2466	0.8720	0.6529	0.049*	
N2	−0.0597 (8)	0.8600 (6)	0.5442 (5)	0.0296 (18)	
H2A	−0.1532	0.8541	0.5371	0.036*	
N3	0.0630 (10)	0.8739 (7)	0.8836 (6)	0.046 (2)	
H3B	0.1305	0.8813	0.9333	0.055*	
C1	0.0159 (10)	0.8685 (8)	0.4712 (7)	0.034 (2)	
C2	0.1627 (10)	0.8756 (8)	0.5147 (7)	0.039 (3)	
C3	0.2710 (12)	0.8833 (10)	0.4600 (8)	0.066 (4)	
H3A	0.3700	0.8858	0.4880	0.079*	
C4	0.2242 (12)	0.8869 (9)	0.3637 (9)	0.061 (4)	
H4A	0.2924	0.8938	0.3253	0.073*	
C5	0.0769 (12)	0.8804 (8)	0.3231 (7)	0.049 (3)	
H5A	0.0494	0.8827	0.2577	0.058*	
C6	−0.0300 (11)	0.8709 (8)	0.3738 (7)	0.041 (3)	
H6A	−0.1284	0.8662	0.3448	0.049*	
C7	0.0345 (9)	0.8625 (7)	0.6271 (7)	0.030 (2)	
C8	−0.0055 (10)	0.8596 (7)	0.7183 (7)	0.030 (2)	
C9	0.1021 (11)	0.8701 (8)	0.8005 (7)	0.042 (3)	
H9A	0.2003	0.8745	0.7970	0.051*	
C10	−0.0742 (12)	0.8670 (8)	0.8940 (7)	0.041 (3)	
H10A	−0.0956	0.8702	0.9539	0.050*	
C11	−0.1834 (11)	0.8552 (8)	0.8174 (7)	0.041 (3)	
H11A	−0.2800	0.8491	0.8243	0.049*	
C12	−0.1504 (10)	0.8524 (7)	0.7299 (7)	0.032 (2)	
H12A	−0.2255	0.8457	0.6771	0.039*	

### Atomic displacement parameters (Å^2^)

**Table:**

	*U*^11^	*U*^22^	*U*^33^	*U*^12^	*U*^13^	*U*^23^
Bi1	0.0332 (2)	0.0371 (2)	0.0254 (2)	−0.00200 (18)	0.00251 (15)	0.00118 (18)
Cl1	0.0385 (15)	0.0486 (19)	0.068 (2)	−0.0013 (13)	−0.0025 (14)	0.0145 (15)
Cl2	0.0353 (15)	0.071 (2)	0.0482 (18)	−0.0031 (13)	0.0091 (13)	0.0045 (15)
Cl3	0.0503 (16)	0.0478 (17)	0.0285 (14)	0.0024 (13)	0.0014 (12)	−0.0016 (12)
Cl4	0.0344 (14)	0.072 (2)	0.0317 (14)	0.0069 (13)	0.0062 (11)	−0.0009 (14)
Cl5	0.0567 (17)	0.0524 (19)	0.0308 (15)	−0.0069 (14)	0.0031 (12)	−0.0090 (13)
O1W	0.057 (5)	0.044 (5)	0.074 (6)	−0.010 (4)	−0.001 (4)	0.022 (4)
N1	0.033 (5)	0.051 (6)	0.034 (5)	0.001 (4)	−0.004 (4)	0.002 (4)
N2	0.022 (4)	0.036 (5)	0.029 (5)	0.005 (3)	0.001 (4)	−0.001 (4)
N3	0.050 (6)	0.053 (6)	0.031 (5)	−0.002 (5)	−0.003 (4)	0.001 (4)
C1	0.032 (6)	0.035 (6)	0.033 (6)	−0.001 (4)	0.002 (5)	0.002 (5)
C2	0.033 (6)	0.049 (7)	0.035 (6)	0.001 (5)	0.007 (5)	−0.002 (5)
C3	0.031 (7)	0.114 (12)	0.052 (8)	−0.002 (6)	0.008 (6)	0.020 (8)
C4	0.043 (7)	0.097 (11)	0.050 (8)	0.005 (6)	0.023 (6)	0.020 (7)
C5	0.052 (7)	0.070 (9)	0.024 (6)	0.003 (6)	0.007 (5)	0.009 (5)
C6	0.039 (6)	0.047 (7)	0.037 (6)	−0.001 (5)	0.009 (5)	0.011 (5)
C7	0.024 (5)	0.028 (6)	0.037 (6)	−0.001 (4)	0.001 (5)	−0.004 (5)
C8	0.043 (6)	0.016 (5)	0.026 (5)	−0.006 (4)	0.000 (4)	0.000 (4)
C9	0.044 (6)	0.048 (7)	0.028 (6)	−0.002 (5)	−0.009 (5)	−0.002 (5)
C10	0.063 (7)	0.032 (6)	0.029 (6)	−0.001 (6)	0.008 (5)	−0.006 (5)
C11	0.050 (7)	0.029 (7)	0.045 (7)	0.001 (5)	0.010 (5)	−0.008 (5)
C12	0.032 (6)	0.024 (6)	0.037 (6)	0.000 (4)	−0.001 (5)	0.003 (5)

### Geometric parameters (Å, °)

**Table:**

Bi1—Cl1	2.519 (3)	C4—H4A	0.9300
Bi1—Cl2	2.624 (3)	C5—C6	1.359 (13)
Bi1—Cl3	2.787 (3)	C5—H5A	0.9300
Bi1—Cl4	2.767 (3)	C6—H6A	0.9300
Bi1—Cl5	2.664 (3)	C7—N1	1.319 (11)
O1W—H1WA	0.8500	C7—N2	1.335 (10)
O1W—H1WB	0.8500	C7—C8	1.443 (12)
N1—H1A	0.8600	C8—C9	1.399 (12)
N2—H2A	0.8600	C8—C12	1.400 (12)
N3—H3B	0.8600	C9—N3	1.327 (12)
C1—C6	1.389 (13)	C9—H9A	0.9300
C1—C2	1.389 (12)	C10—N3	1.322 (13)
C1—N2	1.390 (11)	C10—C11	1.354 (13)
C2—N1	1.376 (12)	C10—H10A	0.9300
C2—C3	1.409 (14)	C11—C12	1.363 (13)
C3—C4	1.375 (15)	C11—H11A	0.9300
C3—H3A	0.9300	C12—H12A	0.9300
C4—C5	1.383 (14)		
			
Cl1—Bi1—Cl2	88.33 (9)	C2—C3—H3A	121.4
Cl1—Bi1—Cl3	97.85 (9)	C3—C4—C5	120.7 (10)
Cl1—Bi1—Cl4	83.99 (8)	C3—C4—H4A	119.6
Cl1—Bi1—Cl5	91.41 (10)	C5—C4—H4A	119.6
Cl2—Bi1—Cl3	84.18 (9)	C6—C5—C4	123.5 (10)
Cl2—Bi1—Cl4	166.23 (9)	C6—C5—H5A	118.2
Cl2—Bi1—Cl5	91.96 (9)	C4—C5—H5A	118.2
Cl4—Bi1—Cl3	85.56 (8)	C5—C6—C1	116.2 (10)
Cl5—Bi1—Cl3	169.84 (8)	C5—C6—H6A	121.9
Cl5—Bi1—Cl4	99.63 (8)	C1—C6—H6A	121.9
H1WA—O1W—H1WB	87.0	N1—C7—N2	107.9 (8)
C2—N1—H1A	124.7	N1—C7—C8	126.9 (9)
C7—N1—C2	110.5 (8)	N2—C7—C8	125.1 (8)
C7—N1—H1A	124.7	C9—C8—C12	116.6 (9)
C1—N2—H2A	125.1	C9—C8—C7	120.1 (9)
C7—N2—C1	109.7 (8)	C12—C8—C7	123.3 (8)
C7—N2—H2A	125.1	N3—C9—C8	119.5 (10)
C9—N3—H3B	118.2	N3—C9—H9A	120.2
C10—N3—C9	123.5 (10)	C8—C9—H9A	120.2
C10—N3—H3B	118.2	N3—C10—C11	120.0 (10)
C6—C1—C2	122.0 (9)	N3—C10—H10A	120.0
C6—C1—N2	132.4 (9)	C11—C10—H10A	120.0
C2—C1—N2	105.5 (8)	C10—C11—C12	119.3 (10)
N1—C2—C1	106.2 (9)	C10—C11—H11A	120.4
N1—C2—C3	133.4 (10)	C12—C11—H11A	120.4
C1—C2—C3	120.3 (10)	C11—C12—C8	121.1 (9)
C4—C3—C2	117.2 (10)	C11—C12—H12A	119.4
C4—C3—H3A	121.4	C8—C12—H12A	119.4

### Hydrogen-bond geometry (Å, °)

**Table:**

*D*—H···*A*	*D*—H	H···*A*	*D*···*A*	*D*—H···*A*
N1—H1A···Cl5^i^	0.86	2.65	3.267 (8)	130
N1—H1A···Cl1^ii^	0.86	2.66	3.302 (8)	133
N2—H2A···O1W^iii^	0.86	1.86	2.680 (9)	159
N3—H3B···Cl4^iv^	0.86	2.33	3.104 (9)	150
O1W—H1WA···Cl3^ii^	0.85	2.62	3.292 (7)	137
O1W—H1WB···Cl5	0.85	2.77	3.190 (7)	112

Symmetry codes: (i) −*x*+1, −*y*+2, −*z*+1; (ii) *x*, −*y*+3/2, *z*+1/2; (iii) *x*−1, *y*, *z*; (iv) *x*, *y*, *z*+1.
